# Predictors of mortality among newborns admitted with perinatal asphyxia at public hospitals in Ethiopia: a prospective cohort study

**DOI:** 10.1186/s12887-021-02779-w

**Published:** 2021-07-07

**Authors:** Samuel Dessu, Zinabu Dawit, Abebe Timerga, Muluken Bafa

**Affiliations:** 1 Department of Public Health, College of Medicine and Health Sciences, Wolkite University, Wolkite, Ethiopia; 2 Department of Nursing, Arba Minch Health Science College, Arba Minch, Ethiopia; 3 Department of Biomedical sciences, College of Medicine and Health Sciences, Wolkite University, Wolkite, Ethiopia

**Keywords:** Perinatal asphyxia, prospective study, Predictor of mortality, Southern Ethiopia

## Abstract

**Introduction:**

Perinatal asphyxia is a complicated newborn health problem and applies a high contribution to the increased proportion of newborn mortality. It occurs in newborns due to altered breathing or inadequate inhalation and exhalation resulting in reduced oxygen perfusion to certain body tissues and organs. Irrespective of the increased progress in health care towards newborns and implementations in reductions in under-five, infant, and neonatal mortality in the past 10 years, perinatal asphyxia remained as the most common severe newborn health challenge that causes a high number of morbidity and mortality.

**Methods:**

A prospective cohort longitudinal study was implemented among 573 newborns admitted with perinatal asphyxia at public hospitals in Southern Ethiopia from 1st March 2018 to 28th February 2020. The perinatal survival time was determined using Kaplan Meier survival curve together with a log-rank test. The dependent variable was time to death and the independent variables were classified as socio-demographic factors, obstetrics related factors, newborn related factors and maternal medical related factors. The study subjects were entered in to the cohort during admission with perinatal asphyxia in the hospital and followed until 7 days of life.

**Results:**

The cumulative proportion of survival among the newborns admitted with perinatal asphyxia was 95.21% (95%CI:91.00,97.48), 92.82% (95%CI:87.95,95.77), 92.02%(95%CI:86.84,95.22) and 90.78%(95%CI:84.82,94.48) at the end of first, second, third and fourth follow-up days respectively. The mean survival date was 6.55(95%CI: 6.33, 6.77) and cord prolapse (AHR:6.5;95%CI:1.18,36.01), pregnancy induced hypertension (AHR:25.4;95%CI:3.68,175.0), maternal iron deficiency anemia (AHR:5.9;95%CI:1.19,29.5) and having convulsion of the newborn (AHR:10.23;95%CI:2.24,46.54) were statistically significant in multivariable cox proportional hazard model.

**Conclusion:**

The survival status among newborns with perinatal asphyxia was low during the early follow-up periods after admission to the hospital and the survival status increased after fourth follow up days. In addition, cord prolapse, history of PIH, maternal iron deficiency anemia and newborns history of convulsion were the independent predictors of mortality.

## Introduction

Perinatal asphyxia is a complicated newborn health problem and applies a high contribution to the increased proportion of newborn mortality [1]. It is a leading cause of morbidity and mortality in newborn babies globally, with higher case fatality rates and consequent complications in developing countries due to poor health facilities [2, 3]. Globally, around 2,500,000 child deaths were reported in the early 28 days of age (neonatal age). These accounts for nearly 47% of under-five mortality and 54% of all under-five deaths occur during this age in developing countries [4].

However, greater than 2/3rd of newborns can be saved through established maternal and newborn health intervention programs. Though, most of the observed deaths have occurred at home delivered newborns [5]. Nearly 3.6 million (3%) of all infants suffer from a certain level of perinatal asphyxia. Among this 840,000 (23%) will die and approximately a similar proportion of newborns develop life-threatening health problems in developing countries [6, 7].

Globally, around 25% of all newborn mortality is caused by perinatal asphyxia [8]. The study conducted at public hospitals in Ethiopia indicated that, perinatal asphyxia contributed to 28.35% of newborn deaths and prematurity and neonatal sepsis accounted for 28.85 and 18.35% respectively [9, 10].

Irrespective of the increased advancements in perinatal care and implementations in reductions of under-five, infant and neonatal mortality in the past decades [4, 11, 12], perinatal asphyxia remains a severe newborn health problem causing a high number of mortality and morbidity and is a major common public health issue, commonly in developing countries like Ethiopia [13].

Even though Ethiopia reached its child mortality reduction goal 2 years earlier, the neonatal mortality rate remained high. One of the major causes of newborn deaths was intrapartum-related complications of which birth asphyxia accounts for 25% [10, 14].

Moreover, a very limited number of studies were conducted in Ethiopia to identify information for intervention regarding the death due to perinatal asphyxia. Therefore; this study was planned to estimate the time to death and its predictors among newborns with perinatal asphyxia at governmental hospitals in Southern Ethiopia.

## Methods

### Study design, setting, period and populations

A prospective cohort longitudinal study was employed at Sawla General Hospital, Arba Minch General Hospital and Chencha district Hospital from first of March 2018 to 28th of February 2020. Among those hospitals, over four thousand newborns were delivered per year and more than 612 newborns were admitted to the neonatal intensive care unit (NICU) at each hospital.

Follow up was initiated at diagnosis of perinatal asphyxia from 1st March 2018 and the follow-up period was closed on 28th February 2020. The study subjects were followed until the age of 7 days and it was closed if the newborn died or censored.

**Fig. 1:**
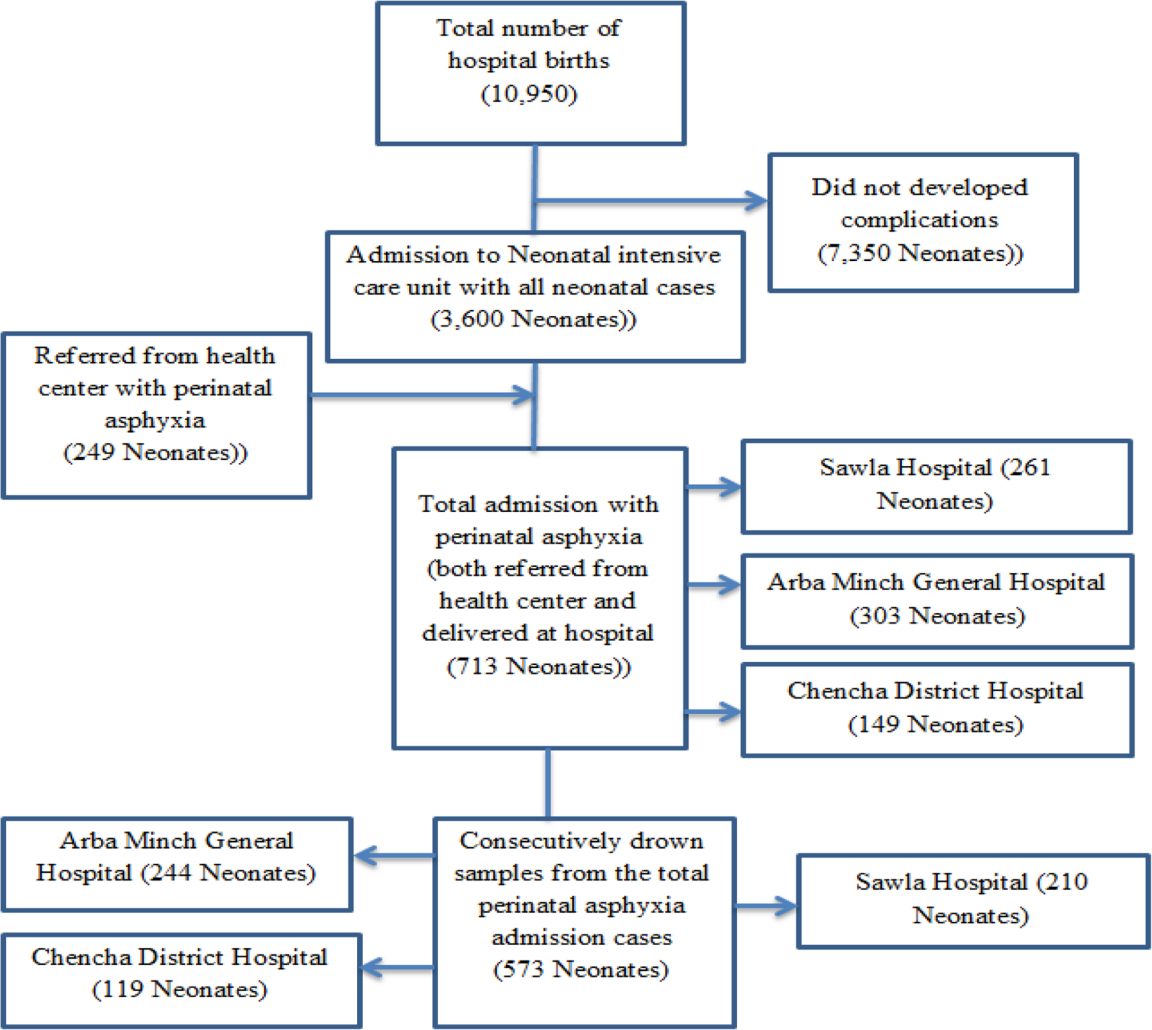
The schematic presentation of sampling procedure for the study on predictors of mortality among newborns presented with perinatal asphyxia at public hospitals at Southern Ethiopia

In this study, a newborn that withdrew treatment, discharged with recovery, transferred to another institution, and who did not yet develop the event at the end of the follow-up period was operationally defined as Censored. Sample size was estimated by Open Epi 3.02 statistical software using double population proportion formula in considering the assumptions; 95%CI, 80% power, exposed to unexposed ratio: 1, percent of unexposed with outcome (Not having history of premature rupture of membrane (PROM)): 50%, percent of exposed with outcome (prolonged labor): 62%, AHR: 1.6 [14] and considering 10% for non-response, the sample size became 573. Sample size was allocated to each hospital proportionally based on the number of the admitted cases and consecutive sampling method was applied **(**Fig. 1).

### Study variables

The dependent variable was time to perinatal mortality and the independent variables were classified as socio-demographic factors (sex of the newborn, maternal age, marital status, a religion of the mother, maternal educational status, maternal occupational status, family size, place of residence, distance between home and hospital and estimated monthly income), obstetrics related characteristics (number of antenatal care (ANC) visits, gravidity, parity, number of pervaginal examinations, history of meconium-stained amniotic fluid, the onset of labor, history of antepartum hemorrhage, history of obstructed labor, history of premature rupture of membrane, history of prolonged rupture of membrane, cord prolapse, presentation of the fetus, mode of delivery and gestational age), newborn related factors (cry immediately at birth, history of convulsion or spasm and birth weight) and maternal medical related characteristics (history of PIH, maternal iron deficiency anemia, maternal diabetes mellitus, and maternal HIV status).

### Operational definitions

Perinatal asphyxia was diagnosed when the newborn had at least one of the following signs: not breathing or gasping, < 30 breaths per minute, or < 7 APGAR score, had neonatal neurologic sequelae (seizures, coma, and hypotonia), or multiple organ involvement (kidney, lungs, liver, heart, and intestines) [10].

Maternal Anemia: Hemoglobin levels of less than 11 g/dl during the first and third trimesters and less than 10.5 g/dl during the second trimester [15].

Premature rupture of the membrane: a rupture (breaking open) of the membranes (amniotic sac) before labor begins [16].

Prolonged rupture of membrane: a rupture of membranes lasting longer than 18–24 h (i.e., between the time of rupture and time of delivery) [16].

Convulsion: newborn who experience an episode of rigidity and uncontrolled jerky motions that generally last a minute or two along with altered consciousness [17].

### Data collection procedure

Structured checklist was used to collect the data. Data extraction tool was carefully designed to improve data quality. In addition; both data collectors and supervisors were trained.

### Data quality control

Pretest was conducted on 29 neonates at Ottona teaching and referral hospital before the actual data collection was initiated and the tool was revised to make it is consistent. The maternal hemoglobin test results were obtained from a laboratory report which was prepared for this research purpose. The hemoglobin level was adjusted for altitude according to criteria set by WHO (World health organization).

### Data processing and analysis

Epi Data version 3.02 was used to enter the data, code the data, edit the data and clean the data. Finally, the data were entered in to Epi Data were exported to SPSS version 25 for statistical analysis. The Kaplan Meier survival curve, together with a log-rank test, was used to estimate the survival time and the time which had higher risk of death. Variables that had a *p*-value < 0.05 in bivariate analysis were considered as candidates for multivariable analysis and variables which had a *p*-value < 0.05 in multivariable cox proportional hazard model were considered as statistically significant.

## Results

### The survival status of newborns with perinatal asphyxia

**Fig. 2:**
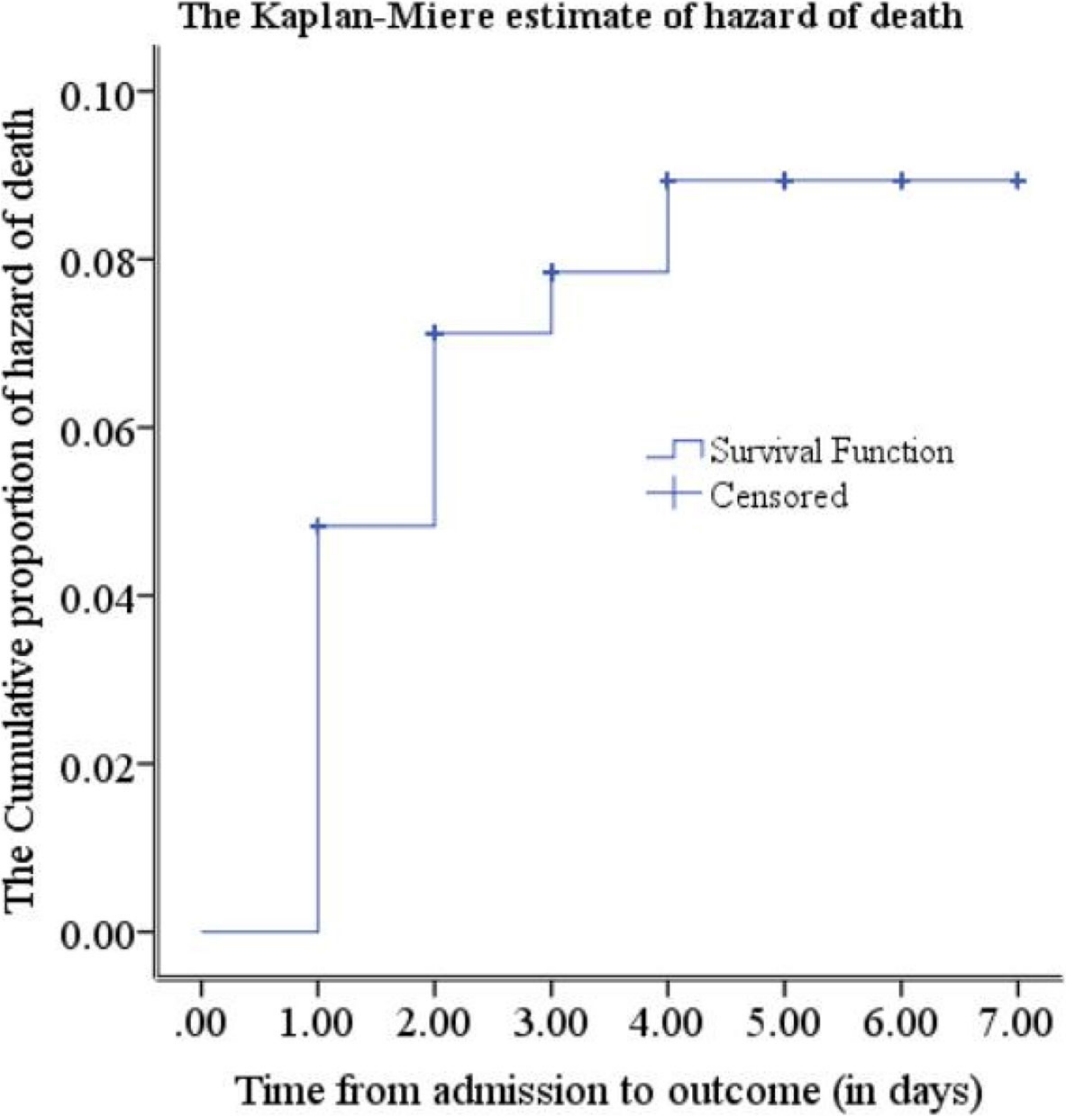
The Kaplan Meier estimate of hazard of death among newborns admitted with perinatal asphyxia

In this study, among a total of 713 newborns, 573 consecutively predetermined samples of newborns were involved. Among them, 45(7.85%) of the newborns with perinatal asphyxia died and 531 (92.67%) were recovered. Among newborns admitted with perinatal asphyxia 27 (4.71%) died in the first follow up day, which is 60% of the observed deaths within the study period. Similarly, the proportion of death at the second and third follow-up days was 2.09 and 0.52% respectively. There was no observed death after the fourth follow-up (Fig. 2).

**Fig. 3:**
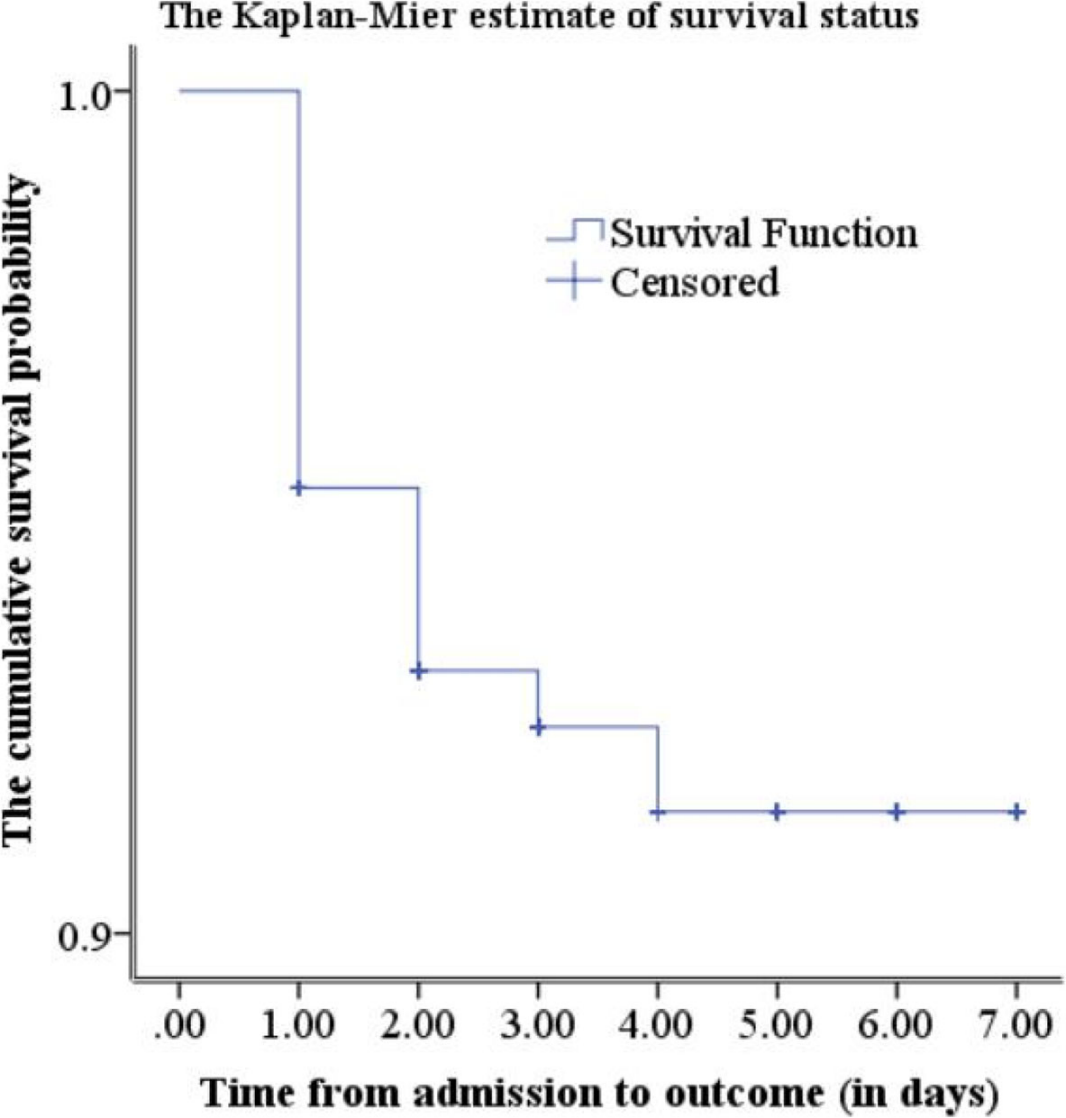
The Kaplan Meier estimate of the survival function among newborns admitted with perinatal asphyxia

The cumulative proportion of survival among the newborns admitted with perinatal asphyxia was 95.21% (95%CI: 91.00, 97.48) at the end of the first follow-up day. In addition, it was 92.82% (95%CI: 87.95, 95.77), 92.02% (95%CI: 86.84, 95.22) and 90.78% (95%CI: 84.82, 94.48) at the end of the second, third, and fourth follow-up days respectively. As we have seen from Fig. 2, there was a rapid decline of survival on the first day and it became slow in the corresponding follow-up days. The overall mean survival time was 6.55 (95%CI: 6.33, 6.77) (Fig. 3).

### Socio-demographic characteristics

In this study a total of 573 newborns were involved, of which 351(61.3%) of them were males. In considering maternal age maximum of the mothers (70.7%) were categorized as under 20–34 years of age and the smallest amount (13.6%) were mothers having age less than 20 years old. Equal numbers of mothers were both unable to read and write and college and above, each accounted for 11% of the whole mothers. Among the dead newborns, 33.3% of mothers were urban residents and 66.7% were rural residents.

In considering the maternal khat chewing and alcohol consumption habits, 18(7.9%) of the mothers had a habit of khat chewing and similar proportions (7.9%) of the mothers had a habit of alcohol consumption. In addition, one newborn was died among the khat chewer mothers, which accounts for 6.7% of the dead newborns. Similarly, one newborn with perinatal asphyxia was died among the mothers who had a history of alcohol intake, which accounts for 6.7% of the dead newborns with perinatal asphyxia (Table 1).

**Table 1:** Socio-demographic characteristics of mothers of the newborn with perinatal asphyxia

Variables	Category	Status of the newborn	
		Died	Survived
		n(%)	n(%)
Sex	Male	27 (60%)	324 (61.4%)
	Female	18 (40%)	204 (38.6%)
Maternal age	< 20	9 (20%)	69 (13.1%)
	20–34	15 (33.3%)	390 (73.9%)
	> 35	21 (46.7%)	69 (13.1%)
Marital status	Not married	30 (66.7%)	21 (4.0%)
	Married	15 (33.3%)	507 (96.0%)
Religion	Orthodox	21 (46.7%)	228 (43.2%)
	Muslim	6 (13.3%)	105 (19.9%)
	Protestant	18 (40.0%)	165 (31.3%)
	Others	0 (0.00%)	30 (5.7%)
Educational status of the mother	Unable to read and write	9 (20.0%)	54 (10.2%)
	Able to read and write	12 (26.7%)	105 (19.9%)
	Grade 1–8	9 (20.0%)	207 (39.2%)
	Grade 9–12	9 (20.0%)	105 (19.9%)
	College and above	6 (13.3%)	57 (10.8%)
Occupational status of the mother	House wife	9 (20.0%)	42 (8.0%)
	Self-employee	12 (26.7%)	120 (22.7%)
	Farmers	9 (20.0%)	198 (37.5%)
	Merchant	9 (20.0%)	117 (22.2%)
	Civil servant	6 (13.3%)	51 (9.7%)
Family size	< 4	9 (20.0%)	264 (50.0%)
	4–6	15 (33.3%)	177 (33.5%)
	> 6	21 (46.7%)	87 (16.5%)
Place of residence	Urban	15 (33.3%)	423 (80.1%)
	Rural	30 (66.7%)	105 (19.9%)
Distance b/n home and hospital	< 10 km	9 (20.0%)	357 (62.3%)
	> 10 km	36 (80%)	216 (37.7%)
Estimated monthly income (ETB)	< 1399	18 (40.0%)	36 (6.8%)
	1400–1999	9 (20.0%)	114 (21.6%)
	2000–2599	9 (20.0%)	189 (35.8%)
	> 2600	9 (20.0%)	189 (35.8%)

### Obstetric related characteristics

In this study, 81(14.1%) of the mothers had no antenatal visits. In addition; 207(36.1%), 87(15.2%), 78(13.6%), and 120(20.9%) of the mothers had one, two, three, and four antenatal visits, respectively. Regarding the number of pervaginal examinations, 351(61.3%) of the mothers had one to three pervagninal examinations. Among the respondents, 465(81.2%), 60(10.5%), 114(19.9%), 129(22.5%) and 120(20.9%) of the newborn’s mother faced the spontaneous onset of labor, obstructed labor, prolonged labor, PROM, and prolonged rupture of membranes, respectively.

Nearly one fifths (21.5%) of the newborns were delivered at the health center. In considering complications during delivery, 102(17.8%) of the newborns with perinatal asphyxia had cord prolapse, and 129(22.5%) of the newborns present with breech presentation. In addition, 408(71.2%), 117(20.4%), and 48(8.4) of the newborns were delivered by SVD and assisted instrumental and cesarean sections, respectively (Table 2).

**Table 2:** Obstetric related characteristics of the newborns with perinatal asphyxia

Variables	Category	Status of the newborn	
		Died	Survived
		n(%)	n(%)
Number of ANC visits	No	18 (60.0%)	54 (10.2%)
	One	9 (20.0%)	198 (37.5%)
	Two	3 (6.7%)	84 (15.9%)
	Three	3 (6.7%)	75 (14.2%)
	Four and above	3 (6.7%)	117 (22.2%)
Gravidity	Primigravida	6 (13.3%)	171 (32.4%)
	Multigravida	39 (86.7%)	357 (67.6%)
Parity	Primipara	3 (6.7%)	144 (27.3%)
	2–4 birth	15 (33.3%)	159 (30.1%)
	Five and above births	27 (60.0%)	225 (42.6%)
Number of pervaginal examinations	1–3	21 (46.7%)	330 (62.5%)
	Four and above	24 (53.3%)	198 (37.5%)
History of Meconium stained amniotic fluid	Yes	36 (80.0%)	78 (14.8%)
	No	9 (20.0%)	450 (85.2%)
Onset of labor	Spontaneous	36 (80.0%)	429 (81.3%)
	Induced	9 (20.0%)	99 (18.8%)
Antepartum hemorrhage	Yes	12 (26.7%)	120 (22.7%)
	No	33 (73.3%)	408 (77.3%)
Obstructed labor	Yes	18 (60.0%)	33 (6.3%)
	No	18 (40.0%)	495 (93.8%)
Duration of labor (hrs)	Less than 18	33 (73.3%)	81 (15.3%)
	Greater than 18	12 (26.7%)	447 (84.7%)
Premature rupture of membrane	Yes	30 (66.7%)	99 (18.8%)
	No	15 (33.3%)	429 (81.3%)
Prolonged rupture of membrane	Yes	33 (73.3%)	87 (16.5%)
	No	12 (26.7%)	441 (83.5%)
Cord prolapse	Yes	33 (73.3%)	63 (13.1%)
	No	12 (26.7%)	459 (86.7%)
Presentation	Cephalic	15 (33.3%)	429 (81.3%)
	Breech	30 (66.7%)	99 (18.8%)
Mode of delivery	Spontaneous vaginal delivery	18 (40.0%)	390 (73.9%)
	Assisted instrumental	21 (46.7%)	96 (18.2%)
	Cesarean section	6 (13.3%)	42 (8.0%)
Gestational age (week)	< 37	12 (26.7%)	279 (52.8%)
	37–42	12 (26.7%)	198 (37.5%)
	> 42	21 (46.75)	51 (9.7%)

### Newborn related characteristics and medical related characteristics of the mother

Nearly half (50.8%) of the newborns were delivered at gestational age, less than 37 weeks and 72(12.6%) of them were post terms (gestational age more than 42 weeks). All of the newborns were resuscitated immediately at birth by trained health professionals and 471(82.2%) of the newborns did not cry during birth. In this study, 165(28.8%), 105(18.3%), 18(3.1%) and 30(5.2%) of the mothers had a history of diagnosed PIH, iron deficiency anemia, DM and HIV respectively (Table 3).

**Table 3:** Newborn related and maternal medical disorders affecting the survival status of the newborns with perinatal asphyxia

Variable	Category	Status of the newborn	
		Died	Survived
		n(%)	n(%)
Cry immediately at birth	Yes	3 (6.7%)	99 (18.8%%)
	No	42 (93.3%)	429 (81.3%)
History of convulsion or spasm	Yes	12 (26.7%)	9 (1.7%)
	No	33 (73.3%)	519 (98.3%)
Birth weight (gram)	< 2500	30 (66.7%)	156 (29.5%)
	> 2500	15 (33.3%)	372 (70.5%)
Pregnancy induced hypertension	Yes	36 (80.0%)	129 (24.4%)
	No	9 (20.0%)	399 (75.6%)
Maternal Iron deficiency anemia	Yes	24 (53.3%)	81 (15.3%)
	No	21 (46.7%)	447 (84.7%)
Maternal Diabetes mellitus	Yes	3 (6.7%)	15 (2.8%)
	No	42 (93.3%)	513 (97.2%)
HIV status	Positive	3 (6.7%)	27 (5.1%)
	Negative	42 (93.3%)	501 (94.9%)

### Log rank estimate of the covariates of variables

The Kaplan Meier survival curve together with the log-rank test estimates the chi square and *p*-value of each variable. Distance between home and hospital, referral status of the newborn, meconium-stained amniotic fluid, obstructed labor, premature rupture of membrane, prolonged rupture of membrane, cord prolapse, presentation, place of delivery, mode of delivery, history of convulsion or spasm, birth weight, pregnancy-induced hypertension, and iron deficiency anemia were candidate variables for multivariable analysis in cox proportional hazard model (Table 4).

**Table 4:** The log rank estimate of the variables determining the survival status among newborns admitted with perinatal asphyxia

Variables	Log rank estimate	
	Chi square (X^2^)	*P*-value
Sex	0.003	0.959
Maternal age	1.365	0.81
Marital status	3.85	0.64
Religion	1.48	0.68
Educational status of the mother	2.97	0.56
Occupational status of the mother	3.73	0.44
Family size	2.21	0.310
Place of residence	1.79	0.32
Distance between home and hospital	20.05	0.0001
Referral status	26.02	0.0001
Estimated monthly income (ETB)	1.89	0.53
Maternal Khat chewing status	0.089	0.765
Alcohol intake	0.031	0.86
Number of ANC visits	3.09	0.74
Gravidity	2.31	0.129
Parity	3.27	0.194
Number of pervaginal examinations	1.43	0.23
Meconium stained amniotic fluid	37.04	0.0001
Onset of labor	0.005	0.94
Antepartum hemorrhage	0.134	0.715
Obstructed labor	43.79	0.0001
Duration of labor	5.43	0.631
Premature rupture of membrane	17.9	0.0001
Prolonged rupture of membrane	28.35	0.0001
Cord prolapse	35.44	0.0001
Presentation	18.40	0.0001
Place of delivery	26.02	0.0001
Mode of delivery	8.36	0.015
Gestational age	0.48	0.487
Cry immediately at birth	1.38	0.239
History of convulsion or spasm	26.32	0.0001
Birth weight (gram)	8.94	0.003
Pregnancy induced hypertension	21.12	0.0001
Maternal Iron deficiency anemia	13.15	0.0001
Maternal Diabetes mellitus	0.733	0.392
Maternal HIV status	0.07	0.79

### The mean survival time among the covariates of predictors of mortality

The mean survival time was different among the covariates of each predictor. The mean survival time was higher among the newborns that had no cord prolapse as compared with those who had cord prolapse during delivery. Similarly, the average survival time was higher among newborns born with a mother who had no pregnancy-induced hypertension and no diagnosed iron deficiency anemia as compared with those with pregnancy-induced hypertension and iron deficiency anemia respectively (Table 5).

**Table 5:** The mean survival date estimate of the newborns with perinatal asphyxia among the covariates of predictors

Variables	Category	Mean survival time (95%CI)
Cord prolapse	Yes	5.19 (4.31, 6.07)
	No	6.85 (6.70, 6.99)
Pregnancy induced hypertension	Yes	5.76 (5.14, 6.38)
	No	6.87 (6.73, 7.01)
Iron deficiency anemia	Yes	5.72 (4.94, 6.50)
	No	6.74 (6.56, 6.93)
Convulsion or spasm	Yes	3.04 (2.07, 4.02)
	No	6.65 (6.45, 6.85)

### Predictors of mortality among newborns with perinatal asphyxia

In this study, cord prolapse, pregnancy induced hypertension, iron deficiency anemia of the mother and having a history of convulsion or spasm of the newborn were statistically significant in the multivariable cox regression model.

Newborns having cord prolapse during delivery had six times higher risk of mortality as compared with those who had no cord prolapse (AHR: 6.5; 95%CI: 1.18, 36.01). The risk of mortality among newborns with perinatal asphyxia and delivered with mothers who had a history of pregnancy induced hypertension was 25 times higher as compared with those who had no pregnancy induced hypertension (AHR: 25.4; 95%CI: 3.68, 175.0).

Newborns with perinatal asphyxia and delivered with mothers with iron deficiency anemia had five times higher risk mortality as compared with those mothers who had no iron deficiency anemia (AHR: 5.9; 95%CI: 1.19, 29.5). Newborns admitted with perinatal asphyxia and had history of convulsion or spasm had 10 times higher risk of mortality as compared with those who had no history of convulsion of spasm (AHR: 10.23; 95%CI: 2.24, 46.54) (Table 6).

**Table 6:** Predictors of mortality among newborns admitted with perinatal asphyxia

Variables	Category	Status		COR(95%CI)	AOR(95%CI)
		Died	Survived		
Distance b/n home and hospital	< 10 km	3	119	1	1
	> 10 km	27	84	8.24 (2.36, 14.12)^a^	2.41 (0.96, 3.86)
Referral status	Yes	11	30	10.52 (3.34, 33.05)^a^	0.65 (0.02, 16.62)
	No	4	146	1	1
Meconium stained amniotic fluid	Yes	12	26	16.95 (4.78, 60.12)^a^	1.46 (0.17, 12.24)
	No	3	150	1	1
Obstructed labor	Yes	9	11	13.76 (4.89, 38.69)^a^	0.48 (0.02, 7.87)
	No	6	165	1	1
Premature ROM	Yes	10	33	7.08 (2.42, 20.72)^a^	1.54 (0.20, 11.90)
	No	5	143	1	1
Prolonged ROM	Yes	11	29	11.34 (3.60, 35.70)^a^	0.50 (0.02, 10.84)
	No	4	147	1	1
Cord prolapse	Yes	11	23	13.68 (4.35, 43.02)^a^	6.5 (1.18, 36.01)^b^
	No	4	153	1	1
Presentation	Cephalic	5	143	1	1
	Breech	10	33	7.22 (2.46, 21.14)^a^	4.1 (0.91, 18.54)
Place of delivery	Health center	11	30	10.52 (3.34, 33.05)^a^	6.4 (0.73, 6.82)
	Hospital	4	146	1	1
Birth weight (gram)	< 2500	10	52	2.09 (1.22, 3.58)^a^	2.99 (0.73, 12.13)
	> 2500	5	124	1	1
Pregnancy induced hypertension	Yes	12	43	3.23 (1.71, 6.09)^a^	25.4 (3.68, 175.0)^b^
	No	3	133	1	1
Maternal Iron deficiency anemia	Yes	8	27	2.28 (1.37, 3.79)^a^	5.9 (1.19, 29.5)^b^
	No	7	149	1	1
Mode of delivery	SVD	6	130	1	1
	Assisted instrumental	7	32	4.2 (1.4, 12.65)^a^	0.87 (0.15, 5.12)
	CS	2	14	2.96 (0.59, 14.70)	5.86 (0.84, 40.77)
Convulsion	Yes	4	3	3.27 (1.84, 5.82)^a^	10.23 (2.24, 46.54)^b^
	No	11	173	1	1

Keynote: ^a^ indicates variables which have *p*-value < 0.25 and ^b^ indicates variables which have *p*-value < 0.05

## Discussion

This study assesses the predictors of mortality among newborns admitted with perinatal asphyxia at public hospitals in Southern Ethiopia and it showed there was a high proportion of mortality at the early admission periods especially at the first day and gradually declines as the follow-up period has been increased.

Newborns having cord prolapse during delivery had six times higher risk of mortality as compared with those who had no cord prolapse (AHR: 6.5; 95%CI: 1.18, 36.01). This study finding is similar with the study conducted in Karachi, Pakistan [2]. The principal causes of perinatal asphyxia in this context was thought to be cord compression and umbilical arterial vasospasm which prevents venous and arterial blood flow to and from the fetus. In addition; it can predispose other factors that lead the newborn to die such as assisted ventilation requirement, low cord pH, meconium aspiration, hyaline membrane disease, convulsion, neonatal encephalopathy, and cerebral palsy [16, 18].

Consistent with the study conducted at Dilla University referral hospital, Southern Ethiopia, Tigray regional state, Ethiopia and tertiary care center in Ahmedabad, Gujarat, India and Ayder comprehensive specialized hospital, Northern Ethiopia [19–22], the risk of mortality among newborns with perinatal asphyxia and delivered with mothers who had a history of pregnancy induced hypertension was 25 times higher as compared with those who had no pregnancy-induced hypertension (AHR: 25.4; 95%CI: 3.68, 175.0). This might be related to the effect of diminished uteroplacental blood flow and placental ischemia, which can be due to pregnancy-induced hypertension which reduces blood flow to the fetus [23, 24]. In addition; pregnancy-induced hypertension (PIH) has an effect on the reduction of blood supply, nutrients, and oxygen to the fetus at intrauterine life, finally which ends up in intrauterine growth restriction. This condition can contribute to newborn mortality with perinatal asphyxia [25].

Newborns with perinatal asphyxia and delivered by mothers who have iron deficiency anemia had five times higher risk mortality as compared with the counterparts who had no iron deficiency anemia (AHR: 5.9; 95%CI: 1.19, 29.5). This study finding is concise with the study done at Dilla University referral hospital, Southern Ethiopia, Southern Nations Nationalities and Peoples Regional State of Ethiopia and Jimma Zone, Southwest Ethiopia [16, 26, 27].

Newborns admitted with perinatal asphyxia and had a history of convulsion of spasm had a 10 times higher risk of mortality as compared with those who had no history of convulsion of spasm (AHR: 10.23; 95%CI: 2.24, 46.54). The possible reason might be convulsion that may cause the newborn to cease breathing (apnea). If this interruption in breathing persists, it can result in a decline in oxygen saturation in the blood to a life-threatening level.

Irrespective of the study conducted at Southern Nepal, Ayder comprehensive specialized hospital, Dilla University referral hospital, primiparity, place of delivery, multi-parity, low birth weight, mode of delivery and premature rupture of the membrane was not statistically significant predictors of mortality [19, 28–30].

### Limitation of the study

Since the study was conducted among the newborns delivered at public hospitals, it cannot be generalized for the newborns delivered at home, health centers and health posts. In addition, this study did not assess the complications secondary to perinatal asphyxia.

## Conclusion

The survival status of newborns admitted with perinatal asphyxia was low at the early follow up periods after admission to the hospital and the survival status improved at the later follow up periods. In addition, having cord prolapse of the newborn during delivery, maternal history of pregnancy-induced hypertension, maternal history of iron deficiency anemia and newborn history of convulsion or spasm were the independent predictors of mortality.

## Data Availability

The data sets generated and/or analyzed are available with a reasonable request through the corresponding author.

## Abbreviations

AHR: Adjusted hazard ratio
ANC: Antenatal Care
APGAR: Appearance, pulse, Grimace, Activity and Respiratory effort
APH: Antepartum hemorrhage
CHR: Crude hazard ratio
DM: Diabetes mellitus
PIH: Pregnancy induced hypertension
WHO: World Health Organization
